# Shear-induced ordering in liquid microjets seen by x-ray cross correlation analysis

**DOI:** 10.1063/4.0000038

**Published:** 2020-10-16

**Authors:** V. Markmann, M. Dartsch, J. Valerio, L. Frenzel, I. Lokteva, M. Walther, F. Westermeier, G. Grübel, F. Lehmkühler

**Affiliations:** 1 Deutsches Elektronen-Synchrotron DESY, Notkestraße 85, 22607 Hamburg, Germany; 2 The Hamburg Centre for Ultrafast Imaging (CUI), Luruper Chaussee 149, 22761 Hamburg, Germany

## Abstract

We applied shear to a silica nanoparticle dispersion in a microfluidic jet device and observed direction-dependent structure along and across the flow direction. The asymmetries of the diffraction patterns were evaluated by x-ray cross correlation analysis. For different Rayleigh nozzle sizes and shapes, we measured the decay of the shear-induced ordering after the cessation of the shear. At large tube sizes and small shear rates, the characteristic times of the decay become longer, but Péclet-weighted times do not scale linearly with Péclet numbers. By modeling particle distributions with the corresponding diffraction patterns and comparing measured shape asymmetry to simulations, we determined the variation of volume fraction over the azimuthal angle for the maximum ordered state in the jet.

## I. INTRODUCTION

The control of a complex liquid sample system by microfluidic jet devices has become of increasing scientific and technological interest in the last few decades, especially at Free Electron Laser (FEL) facilities.^1–5^ The applications include the production of supercooled liquids by evaporative cooling of *μ*m-sized droplets^6–8^ and sample delivery schemes for materials sensitive to radiation damage.^9–11^ Free flowing jets as sample environment have the advantage of a self-refreshing sample and lack of solid boundaries, but low sample volumes often dictate small flow rates and, therefore, *μ*m-thin jets. The shear rates observed in thin liquid jets are in the regime of $\dot{\gamma }\approx 10^{5}$ s^−1^ and, thus, several orders of magnitude higher than in conventional rheometer geometries.^12–14^ However, studies of the influence of shear within the nozzles or the gas environment of the jet are rare.^15^ Higher shear typically leads to more pronounced structure development^16,17^ and, therefore, has to be taken into consideration for time-dependent and complex samples such as biological molecules that are measured in the flow of a liquid jet. Also in spectroscopy, the onset of structure formation may influence the measured signal.^18^ In ultrathin liquid sheets or flat-jets, infrared and soft x-ray spectroscopy becomes possible despite the strong absorption in this regime,^19^ but the small number of molecules in thin jets is especially susceptible to shear-induced alterations in the concentration distribution.

In many liquid jet applications, the influence of shear on the studied particles or molecules is typically disregarded. In order to show the effects of shear, we studied different designs of Rayleigh jet devices.^20^ Unlike gas dynamic virtual nozzles,^21,22^ where a gas flow envelops a liquid jet and compresses it, in Rayleigh jets, the shear is due to the flow profile inside the nozzle. Rayleigh jets are formed upon the rapid exit of a fluid from a nozzle, followed by the subsequent breakup into droplets. In order to obtain a more detailed understanding of time- and space-resolved rheology of colloidal dispersions in a Rayleigh jet, we applied small angle x-ray scattering (SAXS) and scanned a *μ*m-sized beam along a several micrometer thick liquid jet.

Our recent study^23^ on 100 *μ*m thick jets produced by Rayleigh nozzles has shown shear-induced ordering into co-flowing strings of colloidal particles. The formation of co-flowing layers and hydroclusters is due to imbalances between hydrodynamic and thermodynamic forces, which are associated with shear thinning and thickening processes.^24–26^ However, the influence of jet geometries and the magnitude of shear rates on structure formation remains an open question. In this work, we present the time- and space-resolved investigation of shear-induced order in highly sheared free-flowing systems. We analyze scattering patterns with angle-dependent structure factors via x-ray cross correlation analysis (XCCA) and study shear-induced ordering after the cessation of shear forces at the nozzle tip. We observe that the decay of shear induced local ordering is slowest in 100 *μ*m diameter tubes and that characteristic decay times weighted with the Péclet number become constant at high Pe. Additionally, we simulate microscopic particle arrangements to explain our experimental findings.

## II. METHODS

### A. Sample and experiment

We used colloidal silica particles dispersed in water as a sample system. The particles had low size dispersity (≈12%) with a mean radius *r* = 15 nm and a volume fraction of c = 0.18 (Sigma-Aldrich, Ludox TMA 420859).

The scattering experiment was performed in SAXS geometry at beamline P10, PETRA III, Hamburg, Germany. The two-dimensional SAXS patterns were acquired with v×h=2.5 μm×3.5 μm beamsize^27^ at a photon energy of 8 keV and 5 m sample-detector distance using an EIGER X-4M detector.

**FIG. 1.**
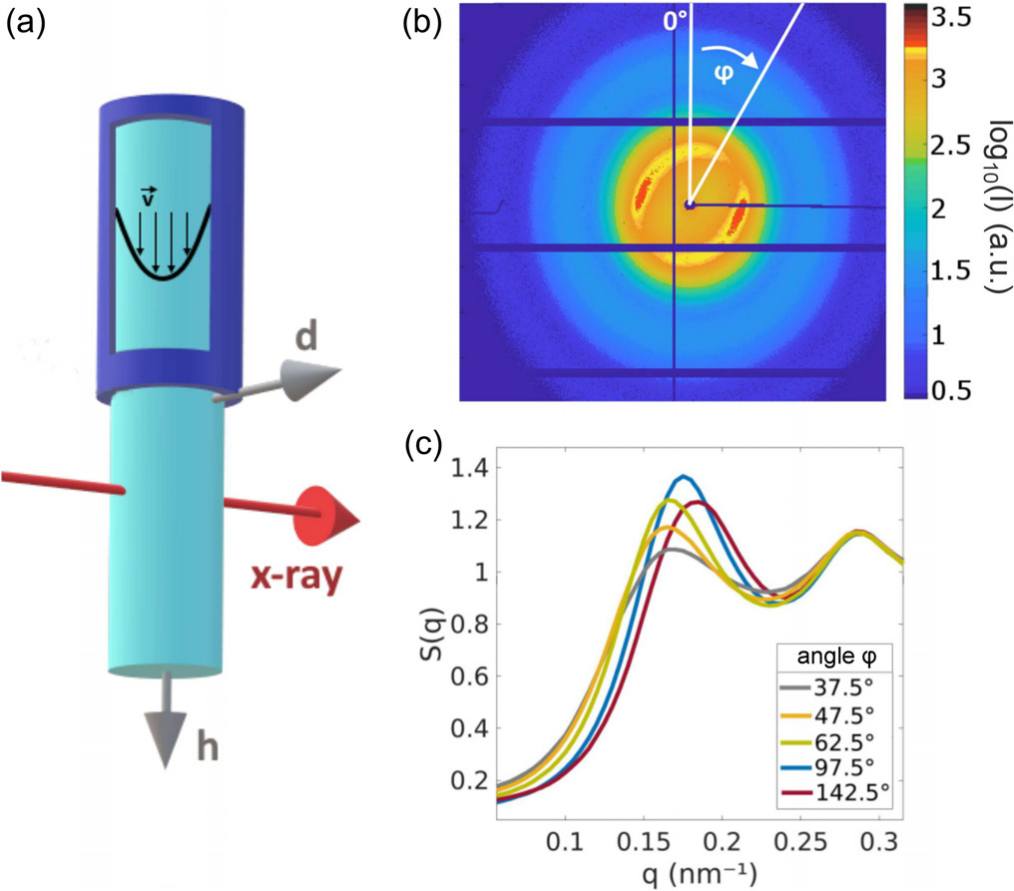
(a) Sketch of the experimental setup. A Rayleigh nozzle produces a jet that is hit by an x-ray beam. The h-axis points vertically downwards along the jet, the d-axis points across the jet, and the third axis runs parallel to the x-ray beam. The resulting scattering pattern in (b) was measured at $\dot{\gamma }=1.9\times 10^{5}$ s^−1^ using a 100 μm square tube, at a nozzle distance h=150 μm and d=0.6·r=30 μm in the jet profile. (c) Structure factor *S*(*q*) over *q* for different azimuthal angles φ for the pattern in (b).

Polyimide coated microtubes (Polymicro Technologies) with diameters of 75 μm, 100 μm, and 150 μm were used as nozzles. A system of four syringe pumps pressed the liquid sample through the tubes as well as recollected the sample from a collecting vessel. Additionally to the cylindrical micro tubes, we used square microtubes with 100 μm edge length. The tubes were placed on the top of a custom-made sample chamber (modified from previous studies^28^). A collecting vessel at the bottom enables the installation of a recycling system for the sample. A sketch of a round nozzle together with a flow profile $\vec{v}$ inside the nozzle, a jet, and a definition of axes used in this work is shown in Fig. 1(a). For reduction of background scattering, the chamber was flushed with a continuous flow of helium during the measurement.

The effective shear rate applied to the sample in round Rayleigh nozzles for Newtonian fluids is given by

$${\dot{\gamma }}_{\mathrm{round}}=\frac{8v_{\mathrm{jet}}}{d_{\mathrm{jet}}},$$ (1)

with the jet velocity *v_jet_* and the jet diameter *d_jet_*.^23,29^ For square tubes, the shear rate was analytically approximated^30^ to

$${\dot{\gamma }}_{\mathrm{square}}=\frac{v_{\mathrm{jet}}}{W}\cdot 7.1136,$$ (2)

with edge length W. We varied the flow rates of the sample between 800 and 2300 μl/min; thus, shear rates between $\dot{\gamma }=0.9\;\text{and}\;5.6\times 10^{5}$ s^−1^ were achieved. The jet lengths for these flow rates were found to be 2–3 mm before breaking up into droplets. We scanned the jets in the jetting regime from the point of emission from the nozzle down to 1250 μm distance as well as horizontally across the jet in 8 μm steps.

### B. X-ray cross correlation analysis

Information about flow-induced local ordering of particles is determined by x-ray cross correlation analysis (XCCA). Therein, the intensity I(q)=I(q,φ) measured by the 2D detector is correlated for different azimuthal angles φ at a given modulus of the wave vector transfer |q|=q=4π sin (θ/2)/λ, where *θ* denotes the scattering angle.^31^ The correlation function,

$$C(q,\Delta )=\frac{{\left\langle I(q,\varphi )I(q,\varphi +\Delta )\right\rangle }_{\varphi }-{\left\langle I(q,\varphi )\right\rangle }_{\varphi }^{2}}{{\left\langle I(q,\varphi )\right\rangle }_{\varphi }^{2}},$$ (3)

describes the orientational order of the sample^32^ with Δ being the angular difference between the two correlated intensities. Via the Wiener–Khinchin theorem, the correlation function C(q,Δ) is connected to the Fourier coefficient ${\hat{I}}_{l}(q)$ of *I*(*q*) via ${\hat{C}}_{l}={\left|{\hat{I}}_{l}\right|}^{2}$, where *l* denotes the symmetry.^33,34^

XCCA can identify orientational order *in situ*, revealing intermediate steps of crystal growth by analyzing emerging Bragg reflections.^35,36^ For non-crystalline materials, a cross correlation shows localized particle ensembles and direction dependent ordering. XCCA has recently been used to identify fourfold and sixfold symmetries in self-assembled thin-films of gold nanoparticles^37^ or the anisotropic preferred orientation of laser pumped metal complex molecules.^38^ In this experiment, we applied XCCA to determine the degree of anisotropy in the scattering patterns.

## III. RESULTS AND DISCUSSION

### A. SAXS characterization

The characteristic SAXS pattern shown in Fig. 1(b) was acquired at d=30 μm in a 100  μ m jet thick jet at h=150 μm. Patterns recorded opposite from the jet center at d=−30 μm are axisymmetrical. Qualitatively similar results were reproduced for all measured shear rates, tube sizes, and tube shapes. Such asymmetrical scattering patterns have been reported previously^23^ for a nozzle distance h=100 μm and $d=0.6\cdot r_{\mathrm{jet}}$. From the asymmetric scattering pattern, the structure factor *S*(*q*) was extracted as an angle-dependent effective structure factor S(q,φ)=I(q,φ)/*P*(*q*). The form factor *P*(*q*) was measured with an unsheared diluted sample (*c* < 0.01) in a glass capillary. The intensity I(q,φ) has been azimuthally integrated with a width of Δφ=5°. Due to Friedel symmetry, we obtain I(q,φ)=I(q,φ+π). Five structure factor peaks at different φ from the scattering pattern are shown in Fig. 1(c). The maximum of *S*(*q*) shifts in *q* between $q_{0}$ = 1.6–1.9 nm^−1^ as well as in peak height with the maximum being observed at $q_{0}=1.75$ nm^−1^. For d≥0 (on the right jet side), the highest *S*(*q*) peak was observed for an angle of φ=97.5° for d≤0 at φ=180°−97.5°=82.5°. These results resemble our previous study,^23^ implying non-isotropic ordering in some regions of the jet. The lowest *S*(*q*) peak was observed for φ=55° (d≥0) and φ=125° (d≤0). Note that the static structure factor of an unsheared sample is in between the minimum and maximum of the angular dependent S(q) in flow, both with respect to peak height and peak position.

### B. XCCA results

**FIG. 2.**
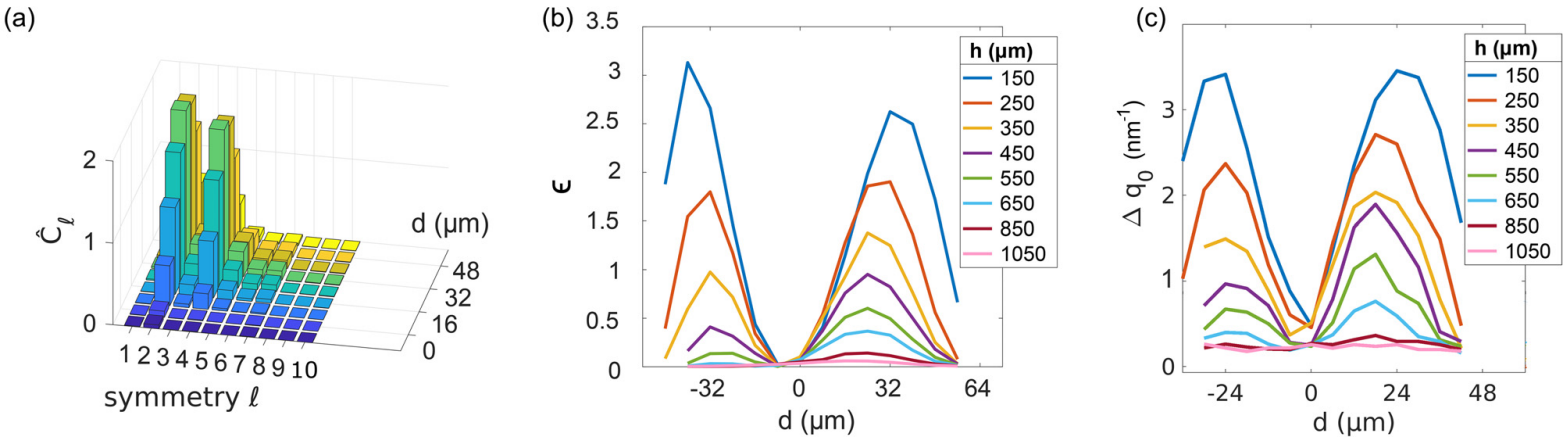
(a) Symmetries ${\hat{C}}_{l}$ over d≥0 (from the center to the right side of the jet) at $q=0.17\;nm^{-1}$. The measurement was performed at h=150 μm distance from the nozzle tip at $\dot{\gamma }=1.7\times 10^{5}$ s^−1^. (b) The contributions to $\epsilon ={\hat{C}}_{l=2}+{\hat{C}}_{l=4}$ for positions *d* across the jet. The color map indicates different distances *h* from the nozzle exit. (c) Shown is $\Delta q_{0}$ over *d* for the same data set as in (a) and (b). $\Delta q_{0}$ represents the square of the standard deviation in the *q*-position of the *S*(*q*) peak over φ.

We investigated the asymmetric behavior of the intensity of the structure factor by means of XCCA. A distribution of Fourier coefficients $\hat{C_{l}}$ for the symmetries *l* = 1–10 taken from the center to the right edge of the jet (*d* = 0–48 μm) is shown in Fig. 2(a) for $\dot{\gamma }=1.7\times 10^{5}$ s^−1^ with a 100 μm square nozzle. The dominating contribution from *l* = 2 and *l* = 4 in the data reflects the twofold symmetry of the non-isotropic scattering pattern. Therefore, the degree of asymmetry in I(q,φ) was studied by the change of $\epsilon ={\hat{C}}_{l=2}+{\hat{C}}_{l=4}$. Other symmetries do not contribute significantly.

The appearance and disappearance of asymmetric patterns was studied extensively by scanning horizontally and vertically over the jet. The asymmetry *ϵ* for positions *d* across the jet is shown in Fig. 2(b). In the jet center, the value of intensity asymmetry *ϵ* drops close to zero and then rises toward the jet edge. Close to the nozzle exit, the first measurement at h=150 μm shows the highest variation in *ϵ* over *d*, and the maxima appear at d=0.6·r=30 μm and d=−30 μm. After an increase in distance *h* of several 100 μm, the structure factor peak maximum shifts slightly towards the jet center before the structure factor peak becomes symmetric in φ and the asymmetry plot of *ϵ* flattens. Additionally to the intensity variations obtained by XCCA, we analyzed the q-position *q*_0_ of the structure factor maximum. The shift of *q*_0_ in φ leads to the oval shape of the scattering pattern and is plotted for every position *d* across the jet in Fig. 2(c), where $\Delta q_{0}=\text{var}(q_{0}(\varphi ))$ describes the variance of the vector $q_{0}(\varphi )$. Both methods show similar asymmetric shear-induced behavior across the liquid jet, yet they represent different aspects in the sample system. While the asymmetry of the scattering pattern observed by XCCA in Fig. 2(b) relates a direction-dependent intensity to a φ-dependent particle ordering, the ovality of the diffraction pattern quantified by the direction-dependent variance in *q*_0_ in Fig. 2(c) relates to a change in next-neighbor distance dependent on φ. This indicates that both particle ordering and local distribution are influenced by shear.

**FIG. 3.**
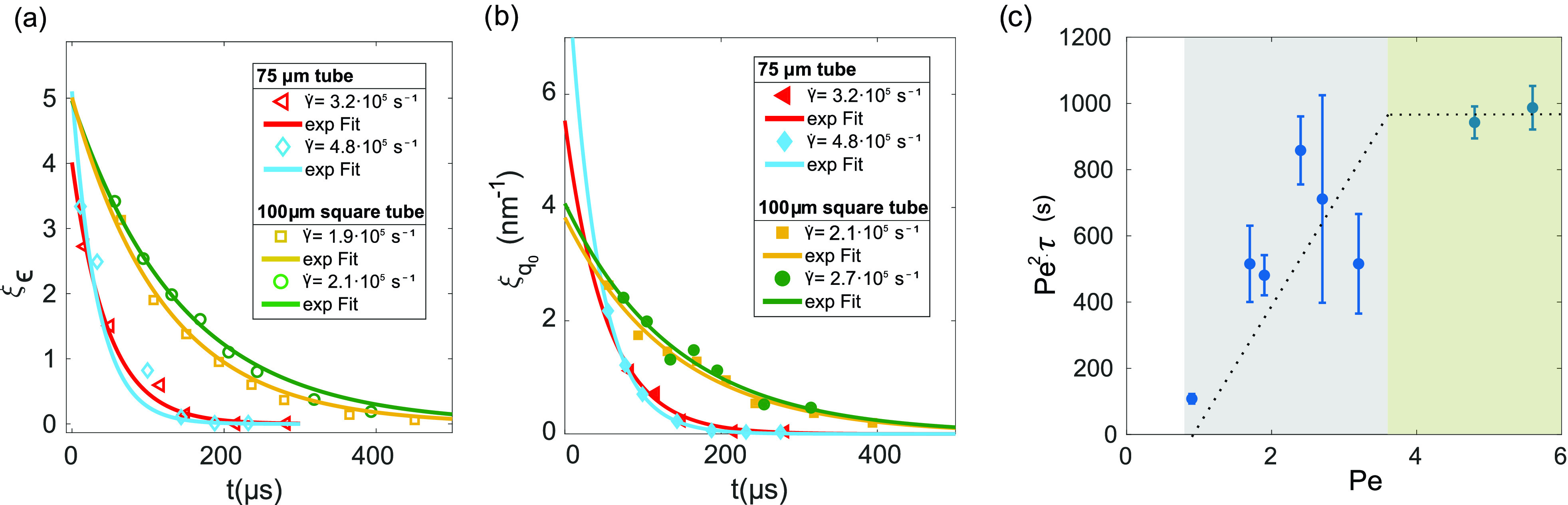
(a) Asymmetry $\xi_{\varepsilon }$ over the time *t* after leaving the nozzle tip [corresponding to Fig. 2(b)]. Shown are four $\dot{\gamma }$ for the 75 μm and 100 μm diameter nozzle. (b) $\xi_{q_{0}}$ over the time *t* [corresponding to Fig. 2(c)]. (c) Characteristic times *τ* extracted from (a) and (b) were weighted with *Pe*^2^. Between *Pe* = 1 and Pe≈3 the increase is linear, for Pe≥3, we observe a transition into constant behavior.

Free-flowing liquid jets enable us to study shear relaxation processes. Therefore, we proceeded to investigate the temporal extent of its decay $\xi_{\epsilon }=\text{max}(\epsilon (h))-\epsilon (h_{\text{max}})$ and $\xi_{q_{0}}=\text{max}(\Delta q_{0}(h))-\Delta q_{0}(h_{\text{max}})$. In addition, we studied the influences of shear rates and nozzle sizes. The decrease in asymmetry of the diffraction patterns with increasing distance in the *h*-axis can be understood with competing interaction, i.e., hydrodynamic interaction and Brownian motion disrupt the shear induced ordering. In Figs. 3(a) and 3(b), we show the exponential decay for 75 μm diameter tubes (red and blue) compared to 100 μm diameter square tubes (yellow and green) for different $\dot{\gamma }$. The decay was fitted with $\xi =a\cdot \;\text{exp}\;(-\frac{t}{\tau })$, where the time *t* was calculated via $t=h/v_{\mathrm{jet}}$ and $v_{\mathrm{jet}}=\frac{Q}{A}$ with the nozzles cross sectional area *A* and the flow rate *Q*. The fitted values of the characteristic times $\tau_{\epsilon }$ and $\tau_{q_{0}}$ for the decay to 1/e (see Table I) are alike within the errorbar; thus, the shear affects both particle order and local distribution in a similar way. The structures developing at high shear rates in small tubes show fast relaxation times; the fastest decay was observed at $\dot{\gamma }=5.6\times 10^{5}$ s^−1^ in the d=75 μm tube at ≤30 μs. Simultaneously, at a larger tube size and small shear rates, the characteristic times become longer. Comparing different tube sizes, both $\xi_{\epsilon }$ and $\xi_{q_{0}}$ decrease faster for the 75 μm and 150 μm tube than for the 100 μm tube at similar $\dot{\gamma }$-values. Shear rate variation up to $\pm 1.6\times 10^{5}$ s^−1^ only slightly influences the exponential trend. For better comparability with shear onset and cessation behavior described in theory studies,^16,39^ the dimensionless parameter $\bar{\tau }\cdot \dot{\gamma }$ was calculated from the weighted mean of $\tau_{\epsilon }$ and $\tau_{q_{0}}$. The highest $\bar{\tau }\cdot \dot{\gamma }$ were found at 29.0 ± 2.3 and 25.1 ± 4.7 for square and round 100  μm tubes, indicating strong and long-lasting structure formation for the studied ratios of jet-thickness to shear rate. When the characteristic times of the different systems are weighted by the square of the Péclet number Pe = $\dot{\gamma }r^{2}/D_{0}$ with *D*_0_, the diffusivity of a particle at radius r, we observe constant behavior above Pe≈3 [see Fig. 3(c)]. This indicates the onset of fast cessation mechanisms at high Pe independent of the system as it has been predicted in theory studies.^16^ In between Pe = 1 and Pe≈3, a linear increase denotes a transition regime between low (Pe≪1) and high Péclet numbers, but further investigation is needed to confirm the trend.

**TABLE I.** Characteristic times of the decay *τ* in *μ*s for $\xi_{\epsilon }$ and $\xi_{q_{0}}$. By multiplying $\dot{\gamma }$ and the weighted mean $\bar{\tau }$ (derived from $\tau_{\epsilon }$ and $\tau_{q_{0}}$), a non-dimensional variable was found for each tube size.

Tube size	d = 150 *μ*m	d = 100 *μ*m	d = 75 *μ*m			$W=d_{\mathrm{square}}=$ 100 *μ*m		
$\dot{\gamma }\;(10^{5}$ s^−1^)	0.9	2.7	3.2	4.8	5.6	1.7	1.9	2.4
$\tau_{\epsilon }$ (*μ*s) for $\xi_{\epsilon }$	122 ± 19	106 ± 114	47 ± 16	34 ± 31	25 ± 7	172 ± 58	120 ± 21	142 ± 18
$\tau_{q_{0}}$ (*μ*s) for $\xi_{q_{0}}$	151 ± 43	91 ± 44	53 ± 28	39 ± 2	30 ± 2	168 ± 50	138 ± 26	142 ± 45
$\bar{\tau }\cdot \dot{\gamma }$	11.4 ± 1.5	25.1 ± 4.7	17.8 ± 0.7			29.0 ± 2.3		

### C. Simulation

**FIG. 4.**
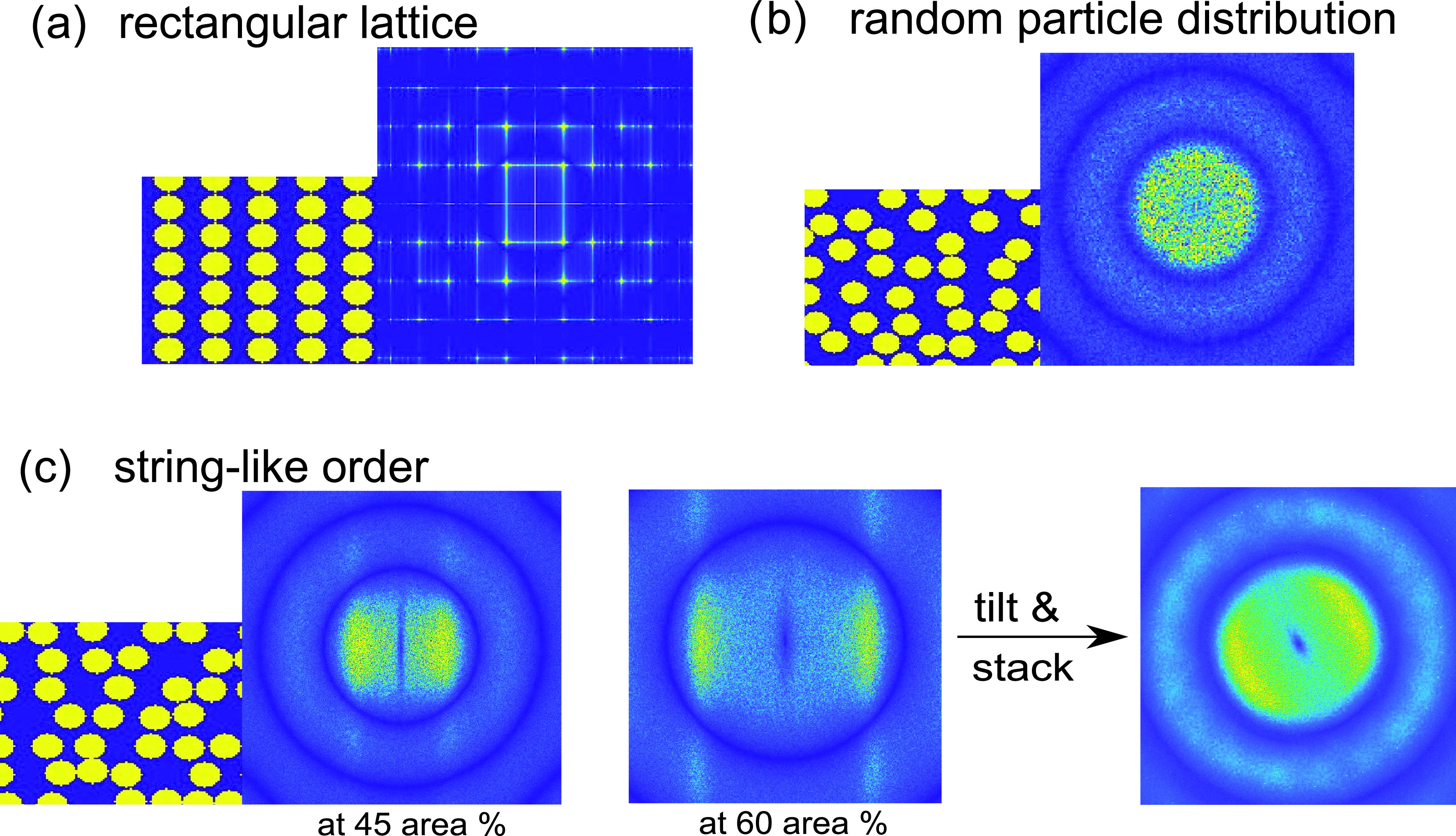
Simulated particle arrangements and diffraction patterns. Via FFT, the diffraction pattern for (a) particles on a rectangular lattice structure (the starting point of the simulation) (b) a random distribution of particles, and (c) particles distributed in string-like order for 45 area% and 60 area% concentration are shown. By turning and overlaying multiple diffraction patterns from string-like particle distributions, an asymmetric diffraction pattern as in the SAXS measurements is obtained.

The experimental data suggest a non-isotropic microscopic particle arrangement in the jet region of asymmetrical scattering. Therefore, we simulated multiple two-dimensional configurations for hard disks in co-flowing string-like order. Hard disks were first placed on rectangular lattice points as shown in Fig. 4(a). Afterwards, disorder was introduced using a Monte Carlo approach, moving the particles to new positions avoiding overlapping of neighboring particles. The random particle distribution in Fig. 4(b) was configured by allowing each particle to move randomly in horizontal and vertical directions. String-like arrangements [Fig. 4(c)] were achieved by limiting degrees of freedom for movements in vertical or horizontal directions to 1/10 of the particle radius and reducing the quantity of steps. To obtain the diffraction patterns, we applied fast Fourier transformation (FFT) to the aforementioned particle arrangements from boxes of 490 000 particles. The resulting asymmetric diffraction patterns for string-like particle arrangements were then tilted and stacked on top of each other in order to rebuild the three-dimensional jet. By arranging only specific angles of co-flowing strings to be used in the stacking, the angle-dependent contributions of the intensity in the diffraction patterns were modified to resemble our measured SAXS data. An exemplary diffraction pattern is shown in Fig. 4(c). The stacking contains four configurations of particles, orientated parallel to the d-axis and sloping down in 20° steps to being parallel with the h-axis, thus creating intensity maxima in the diffraction pattern between 90° and 180°. Experimentally, we found the minimum in peak height of the intensity occurring at φ=55° for d≥0 and at φ=125° for d≤0, so simulating a corresponding diffraction pattern requires a particle formation of co-flowing strings not parallel to the *h*-axis (flow direction) but tilted outwards from the jet center, forming an A-shaped string pattern across the jet. The model used for the simulations does not rule out other possible particle arrangements, but is a qualitative model able to describe our experimental findings.

Ovality of the diffraction pattern was considered to be connected with concentration differences between the stacked patterns and was further analyzed in a second step. Therefore, we extract the angular particle concentration distribution. As the number of particles is given as a parameter for the simulation, we calculate the two-dimensional effective volume fraction for each particle configuration before applying FFT.

**FIG. 5.**
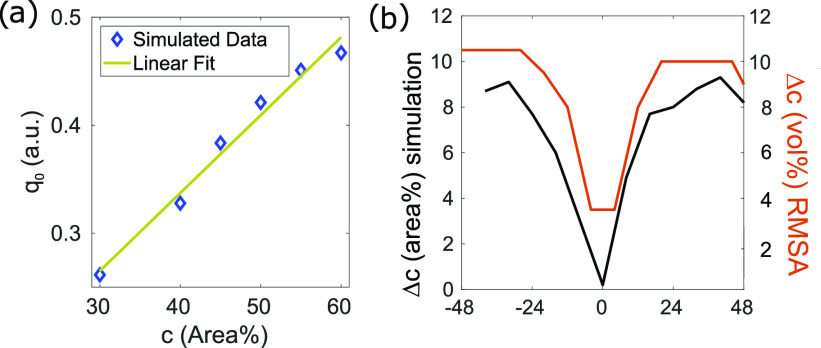
(a) The position of *q*_0_ for different area concentrations is shown for simulated two-dimensional diffraction patterns. (b) Concentration difference Δc across the jet approximated by *q*_0_ positions. The slope of the linear fit in (a) was extracted to convert measured *q*_0_ variances in φ into concentration differences. Additionally, the concentration difference calculated using the RMSA.

The positions *q*_0_ of the structure factor peak in the calculated diffraction pattern show a linear behavior for known area concentrations depicted in Fig. 5(a). From the experimental results, we extract the ratio between the long and short axis of the oval-shaped diffraction patterns. With the slope from the linear fit in Fig. 5(a), we transform the two *q*_0_ positions into a concentration distribution Δc over *d* [Fig. 5(b)].

Additionally, the local, direction-dependent volume fraction has been analyzed with the rescaled mean spherical approximation (RMSA) model^40^ for charge-stabilized spherical particles. We determine angle-dependent volume fractions from the measured diffraction patterns and create a second Δc by subtracting the minimum and maximum concentrations revealed by RMSA [red curve in Fig. 5(b)]. Compared to the results of Δc determined by the axis ratio from the same measured data, the distribution of Δc across the jet from the RMSA fit is in good accordance for both characterizing methods.

**FIG. 6.**
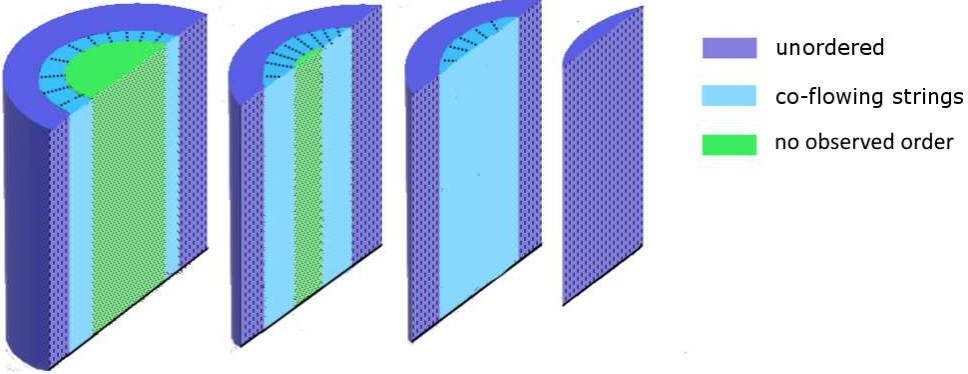
Schematic of the proposed sections of a liquid jet (not to scale). Three regions of different microscopic particle ordering are shown color-coded.

At last, we modeled the jet by presuming three regions for the jet profile, as shown in Fig. 6. Shear forces are applied by the inner walls of the nozzle to the liquid. Flow profiles within small pipes of different geometries have been well studied^41,42^ and show a maximum flow velocity at their center. When the x-rays shine through the center of a liquid jet (green region in Fig. 6), no asymmetric diffraction pattern is observed due to the string formation along the observation axis. Moving out of the jet center increases the scattering volume of string-like formations which are not parallel to the beam direction; therefore, asymmetric structures are observed in diffraction patterns outside of the jet center (light blue region in Fig. 6). At the jet edge, the string-like structure is counteracted by turbulences on the liquid/air interface^43^ and diffusion dominates in areas of slower flow velocity. Both turbulences and diffusion lead to less ordering in the dark blue region in Fig. 6. SAXS measurements at the interface area are dominated by streaks due to scattering from the jet curvature. The dimension of the proposed jet sections needs to be investigated further in future experiments, but the core region with no observed order as well as the unordered outer region seems to be comparably small (≤10 μm) for all nozzle sizes.

## IV. CONCLUSION

We reported detailed studies of the mechanisms in liquid jets after shear cessation. Therein, nozzle sizes, shapes, and shear forces applied by the flow were found to contribute significantly to order formation in the jet. Micrometer resolutions allowed us to reveal relations between shear rate, the decay of shear-induced ordering, and nozzle size. By XCCA, we could assign the parameter $\xi_{\epsilon }$ as the angle-dependent ordering of particles at various jet positions. A second parameter $\xi_{q_{0}}$ describing angle-dependent particle-particle distances shows remarkable similar decreasing behavior over time as $\xi_{\epsilon }$. The coexistence of both phenomena is found in the studied colloidal dispersion independent of other probed parameters such as flow rate or nozzle profile.

The dimension-free parameter $\bar{\tau }\cdot \dot{\gamma }$ compares the decay of ordering in different nozzle sizes and shapes. The highest value was found for the 100  μm square tubes, indicating long-lasting shear effects. Furthermore, the characteristic decay time *τ* weighted by *Pe*^2^ showed a transition to constant behavior at Pe≈3.

The angular variation in intensity of the structure factor peak reveals a preferred orientation of the co-flowing strings tilted in flow direction away from the jet center. This was quantified by simulations of co-flowing string-like arrangements. For the q-position of the structure factor peak, the angular dependence was modeled with varying particle concentrations. From the ratio between the long-and short-axis of the oval-shape diffraction pattern, we approximated the direction-dependent effective volume fraction in our experimental data, which was in good accordance with the localized volume fraction from the RMSA data analysis.

Our results indicate that jet-based sample delivery relies on well understood flow mechanics as well as the impact of shear rate and tube size, which are accessible with this spatial and temporal approach to liquid jet-based rheology. Furthermore, spectroscopy techniques such as photoelectron spectroscopy on liquid jets may influence the signal in regions of strong order in the outer layers of the jet.

## Data Availability

The data that support the findings of this study are available from the corresponding author upon reasonable request.
